# Air Pollution and the Progression of Physical Function Limitations and Disability in Aging Adults

**DOI:** 10.1001/jamanetworkopen.2025.58699

**Published:** 2026-02-11

**Authors:** Jiaqi Gao, Carlos F. Mendes de Leon, Adam A. Szpiro, Jennifer Weuve, Kenneth M. Langa, Richard A. Hirth, Kelly M. Bakulski, Jinkook Lee, Boya Zhang, Jennifer D’Souza, Kayleigh P. Keller, Joel D. Kaufman, Jessica Faul, Sara D. Adar

**Affiliations:** 1 Department of Epidemiology, University of Michigan School of Public Health, Ann Arbor; 2 Department of Oncology, Georgetown University School of Medicine, Washington, DC; 3 Department of Biostatistics, University of Washington, Seattle; 4 Department of Epidemiology, Boston University School of Public Health, Boston, Massachusetts; 5 Institute for Social Research, University of Michigan, Ann Arbor; 6 University of Michigan Medical School, Ann Arbor; 7 Institute for Healthcare Policy and Innovation, University of Michigan, Ann Arbor; 8 Department of Health Management and Policy, University of Michigan School of Public Health, Ann Arbor; 9 Center of Economic & Center for Economic and Social Research, University of Southern California, Los Angeles; 10 Department of Statistics, Colorado State University, Fort Collins; 11 Department of Epidemiology, University of Washington, Seattle; 12 Department of Environmental and Occupational Health Sciences, University of Washington, Seattle; 13 Department of Medicine, University of Washington, Seattle

## Abstract

**Importance:**

Physical disability reflects the cumulative burden of chronic conditions. Although generally progressive, episodes of disability can be followed by periods of recovery; therefore, there is a need to identify modifiable risk factors that contribute to the dynamic development of disability.

**Objective:**

To investigate air pollution as a modifiable risk factor of transitions between states of no physical function limitation, physical function limitations, and activities of daily living (ADL) disability.

**Design, Setting, and Participants:**

This cohort study included respondents older than 50 years from the nationally representative Health and Retirement Study (HRS) who participated in at least 2 interviews between 2000 and 2016. Data analysis was conducted from July 2023 to August 2025.

**Exposures:**

Ten-year average ambient concentrations of particulate matter with a diameter of 2.5 µm or less (PM_2.5_), PM with a diameter between 10 and 2.5 µm (PM_10-2.5_), nitrogen dioxide (NO_2_), and ozone (O_3_) were estimated at respondent residential addresses preceding each survey using spatiotemporal models.

**Main Outcomes and Measures:**

Physical disability states were assessed using self-reported mobility and Activities of Daily Living (ADL). To examine associations of exposure to air pollution with transitioning between states of physical disability, multistate models, adjusted for individual- and area-level covariates, were used.

**Results:**

The sample included 29 790 respondents (mean [SD] age, 63 [11] years; 16 878 [57%] women; 3371 [11%] Hispanic, 5240 [18%] non-Hispanic Black, and 20 314 [68%[ non-Hispanic White), who were followed up for a mean (SD) of 8 (6) years. IQR-increments in PM_2.5_, PM_10-2.5_, and NO_2_ concentrations were mostly associated with greater hazards of transitioning from a state of no physical function limitation toward disability; a 1-IQR increment for PM_2.5_ was associated with lower odds of a reverse transition. For example, in the single-pollutant model, a 1-IQR higher PM_2.5_ concentration was associated with a hazard ratio (HR) of 1.06 (95% CI, 1.03-1.09) for transitioning from no physical function limitations to physical function limitations and an HR of 0.96 (95% CI, 0.93-0.99) for reverting back to healthy physical function from physical function limitations. By contrast, a 1-IQR higher O_3_ concentration was associated with lower hazards of transitioning from no physical function limitations to physical function limitations (HR, 0.92; 95% CI, 0.86-0.98) and ADL disability (HR, 0.89; 95% CI, 0.81-0.97).

**Conclusions and Relevance:**

These findings suggest that air pollution may affect the progression of physical disability and hinder recovery in later life.

## Key Points

**Question:**

Is long-term exposure to air pollution associated with the dynamic physical disability process?

**Findings:**

In this cohort study of 29 790 adults older than 50 years who were followed up for nearly 20 years, higher concentrations of particulate matter with a diameter of 2.5 µm or less (PM_2.5_), PM with a diameter between 2.5 and 10 µm (PM_10-2.5_), and nitrogen dioxide (NO_2_) were associated with a higher risk of transitioning to more severe states of physical disability, and a higher concentration of PM_2.5_ was associated with a lower likelihood of reverting from physical function limitation to healthy physical function. Unexpectedly, higher concentrations of ozone (O_3_) were associated with a lower risk of disability development and progression.

**Meaning:**

These findings suggest that reducing air pollution levels may help to delay and mitigate physical disability in aging adults.

## Teaser

This cohort study evaluates whether exposure to common air pollutants, including particulate matter, nitrogen dioxide, and ozone, is associated with the dynamic development of disability among US adults older than 50 years.

## Introduction

Increasing life expectancies have resulted in shifts from early death to prolonged years lived with various manifestations of physical disability.^1,2^ The burden of physical disability is considerable, with emotional and economic repercussions at both personal and societal levels. For example, adults aged 50 years and older with a physical disability average more than 35 medical visits per year, which is 14 times more than their peers without disability.^3^ Moreover, the average health costs for these adults exceeded $21 000 per year, 57% more than for those without physical disability.^4^ Given these burdens, it is crucial to identify modifiable determinants of physical disability to prevent or delay its onset.

Physical disability is typically developed over a span of many years, following a series of biological states starting with subclinical physiological changes and chronic disease. As these conditions worsen, adults progress to physical function limitations and then disability, as defined by difficulties performing routine activities of daily living (ADL).^5^ Notably, the disability process is not always linear, with periods of physical function limitations or ADL disability sometimes followed by periods of recovery, especially after acute health events or hospitalizations.^4,5^ This suggests the importance of evaluating the disability process as a continuum to identify factors that either exacerbate or reverse this process once it has begun.

Long-term exposure to air pollutants, including particulate matter (PM), nitrogen dioxide (NO_2_), and ozone (O_3_), are well-established risk factors for multiple chronic diseases^6,7,8,9^ and thus are likely contributors to physical function limitations and ADL disability. Exposure to these pollutants can induce systemic inflammation, oxidative stress, and endothelial dysfunction. These changes can then lead to declines in cardiovascular, respiratory, and metabolic health, which are central to maintaining physical mobility and strength.^10,11^ It has also been suggested that air pollution can disrupt endocrine function and directly cause vitamin D deficiency, subsequently leading to poor physical function health due to bone loss.^12^ Collectively, these pathways provide biologically plausible mechanisms through which air pollution can lead to functional limitations and disability, yet research on this topic is fragmented.

Existing studies on air pollutants and physical function limitations^13,14^ or ADL disability^15,16^ generally examine these relationships separately, rather than together as an interconnected process. Relatedly, existing research has focused on the role of air pollution in physical function decline without considering whether it also may hinder the reverse process toward recovery. This potentially limits our understanding of the impact of air pollutants on the dynamic continuum that connects states of no physical function limitations with physical function limitations and ADL disability. Therefore, we extended our previous work within the Health and Retirement Study (HRS)^17^ to characterize associations between long-term exposure to fine (ie, ≤2.5 µm [PM_2.5_]) and coarse (ie, 10-2.5 µm [PM_10-2.5_]) PM, NO_2_, and O_3_ and transitions between states with no physical function limitations, physical function limitations (as an intermediate state of physical disability), ADL disability (the most severe state of physical disability), and death.

## Methods

### Study Population

The HRS is an ongoing, prospective cohort study established in 1992 to investigate factors associated with healthy aging.^18^ Researchers initially enrolled a demographically diverse sample of approximately 20 000 people older than 50 years using a stratified multistage sampling design. To maintain a nationally representative sample, the cohort is refreshed every 6 years. In-person or phone interviews are conducted biennially with each respondent, during which respondents answer an extensive survey covering demographic characteristics, health-related life behaviors and outcomes, and socioeconomic information.

The Environmental Predictors of Cognitive Health and Aging (EPOCH) study added environmental information to the HRS during the period of 2000 to 2016. Therefore, we included all respondents with at least 2 interviews during this time and excluded those with incomplete records on exposure, outcomes, or covariates that were critical for adjustments in our models.

The HRS is sponsored by the National Institutes on Aging and is conducted by the University of Michigan. The HRS and EPOCH studies were approved by University of Michigan institutional review board, and written informed consent was obtained from all study participants. The reporting of this study follows the Strengthening the Reporting of Observational Studies in Epidemiology (STROBE) reporting guideline for cohort studies.

### Physical Function Limitations and ADL Disability Assessments

We defined physical function limitations using data on impaired mobility^19^ based on self-reported difficulty performing any of the following 5 tasks for more than 3 months due to health problems: walking across the room, walking 1 block, walking several blocks, climbing 1 stair, or climbing several stairs. Responses of “can’t do” or “don’t do” were also coded as impairment. In our primary models, we defined physical function limitations as an impairment in walking 1 block and climbing 1 stair. As a secondary outcome, we created a summary score of impairment across all 5items, with a score of 0 indicating no physical function limitations and a score of 5 indicating the highest level of physical function limitations.

We also assessed ADL disability based on self-reported difficulty or needing assistance in performing any of the following 6 tasks for more than 3 months due to health problem: bathing, eating, walking, dressing, toileting, and getting in and out of bed. As a secondary outcome, we generated a summary score by counting all difficulties across the 6 tasks, with a higher score indicating more severe ADL disability. Mortality information, including the date of death, was reported by proxy respondents.

### Residential Exposure Assessment

In EPOCH, we estimated time-weighted concentrations of outdoor PM_2.5_, PM_10-2.5_, NO_2_, and O_3_ at each respondent’s home during the exact 10-year period preceding each survey using spatiotemporal modeling approaches described previously.^20,21,22^ Briefly, the models for this study were constructed to estimate annual average (1990-1998) or 2-week average (1999-2016) levels of each pollutant at exact locations and were calculated for the home addresses of all HRS participants using their residential histories. These models use data from the US Environmental Protection Agency Air Quality System and several regional research studies, over 300 geographical covariates (eg, local emission sources, population density, and land use), and spatial and temporal correlation. The cross-validated accuracy of these models ranged from *R*^2^ of 0.6 to 0.9.

We calculated averages of PM_2.5_, PM_10-2.5_, NO_2_, and O_3_ concentrations for each respondent time-weighted to their residential history based on their survey date. While our primary exposure metric was a 10-year moving average before each interview, we also estimated 5-year and 1-year averages for sensitivity analyses.

### Covariates

In our analyses, we incorporated a wide range of personal demographic and socioeconomic covariates gathered by HRS during each interview. These included age, sex (female or male), race and ethnicity (Hispanic, non-Hispanic Black, non-Hispanic White, and additional groups [ie, those with race and ethnicity other than Black, Hispanic, or White]), education level (less than high school, General Equivalency Diploma, high-school graduate, some college, college and above) as well as information on primary residence ownership (yes or no) and household baseline net wealth. We also estimated neighborhood socioeconomic status (NSES) for respondents’ home locations and 11 census variables^23^ where higher scores indicate higher NSES. Since respondents come from the entire continental United States, we also utilized urbanicity and spatial-basis spline with 10 degrees of freedom^24^ to address spatial confounding. Urbanicity was categorized at the county level as urban (>1 million people), suburban (250 000 to 1 million people), and rural (<250 000 people), using the Beale Rural-Urban Continuum Codes.^25^

### Statistical Analysis

**Figure 1.**
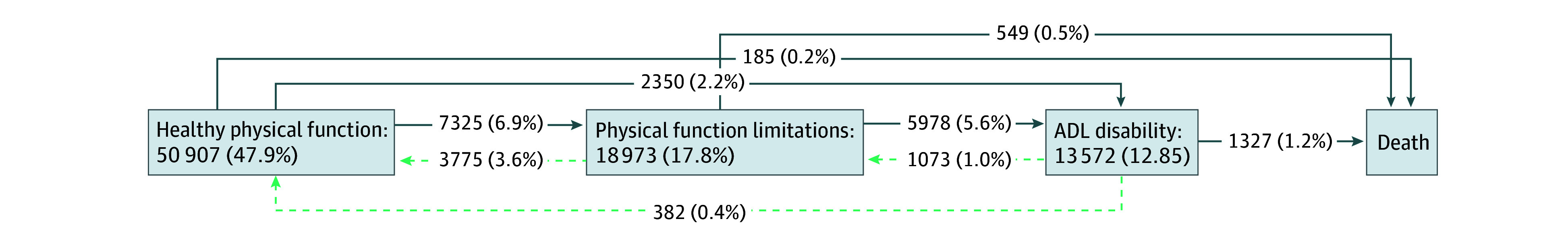
Modeled States of Physical Function Limitations and Disability and Counts From Paired Examinations During Follow-Up in the Health and Retirement Study (2000-2016) Solid lines represent the progression from healthy to physical function limitations, activities of daily living (ADL) disability, and death. Dashed lines represent the reverse process between ADL disability, physical function limitations, and health. Death is the absorbing state. The values in the boxes represent the participants who did not transition between adjacent waves; the values with each line represent those who did transition.

We first characterized the distribution of key characteristics of all participants at baseline based on physical function state and explored correlations between pollutants. We then implemented a multistate model to characterize how long-term exposures to air pollution are associated with transitions between each state of physical disability,^26^ defined as without physical function limitations, any physical function limitations, any ADL disability, and death as the absorbing state (Figure 1). The model estimated hazards for all observed transitions within the full cohort simultaneously, including both adjacent transitions (eg, healthy to physical function limitation) and nonadjacent transitions (eg, healthy to ADL disability). The model also allows transitions in both directions and multiple transitions within an individual over the course of their follow-up. We assumed time-homogeneous transition rates after adjustment for covariates, which is consistent with prior applications of continuous-time multistate models in aging research and provides a stable estimation across irregular interview intervals.

We adjusted all models for time (baseline age, years in study, calendar year of examination, which together capture time, age, cohort, and birth cohort effects), demographics (sex, race and ethnicity), socioeconomic status (education level, household baseline net wealth, primary residence ownership, and NSES), and spatial covariates (urbanicity and spatial basis spline derived from the participant’s residential address with *df* of 10). These covariates were selected a priori as potential confounders. We also examined confounding by copollutants by estimating 2- and multiple-pollutant models. All models were scaled to an IQR (ie, 75th percentile − 25th percentile) for comparability across pollutants. Given that the main aim of the study is to identify the determinants of physical disability process, with death being the absorbing state, we only present the transition hazards between no physical function limitations, physical function limitations, and ADL disability.

In secondary analyses, we also employed generalized estimating equations (GEEs) to separately assess the associations between long-term air pollution exposures and the progression of physical functional limitations and ADL disability over time as continuous measures. In these models, we simultaneously estimated associations of air pollution with our 2 summary scores at baseline and as a rate of change over time. To do so, we included a fixed effect for concentrations preceding the baseline for the cross-sectional component of the model and an interaction between the average concentration over the follow-up period and follow-up time for the rate of change.^27^ Our primary interests were associations with the rate of change in the physical function limitations and ADL disability after adjustment for the covariates specified previously.

We performed sensitivity analyses to test the robustness of our models. First, we refitted the multistate model among respondents who were free of either physical function limitations or disability at baseline. Second, we repeated both the multistate and GEE models with 1-year and 5-year average exposures. All analysis were conducted using the msm package^28^ in R version 4.1 (R Project for Statistical Computing) and proc genmod in SAS version 9.4 (SAS Institute). Statistical significance was set at *P* < .05, and analyses were conducted from July 2023 to August 2025.

## Results

A total of 29 790 respondents (mean [SD] age, 63 [11] years) met our inclusion criteria and were followed up for a mean (SD) of 8.0 (5.9) years (Table; eFigure 1 in Supplement 1). Characteristics of respondents excluded and included in the study appear in the eTable in Supplement 1. There were 16 878 (57%) female participants; 3371 (11%) Hispanic, 5240 (18%) non-Hispanic Black, and 20 314 (68%) non-Hispanic White participants. Most were from an urban area (23 976 [80%]) and had at least some college education (12 588 [42%]) (Table). At baseline, the mean (SD) number of physical function limitations was 1.0 (1.5), whereas most people did not report any physical disability (mean [SD] number of ADL difficulties, 0.3 [0.9]). Across a total of 106 306 unique examination pairs (Figure 1), we observed that respondents most commonly remained in the same physical function state (50 907 [48%] healthy, 18 973 [18%] with physical function limitations, and 13 572 [13%] with ADL disability). Progression events occurred during 15 653 observations (15%), with the most observed in transitions from healthy to physical function limitations and physical function limitations to ADL disability. Reversions were less frequent between adjacent waves that were 2 years apart (5230 observations [5%]), and the most common reversion occurred from physical function limitations back to healthy.

**Table.** Demographic Characteristics of Health and Retirement Study Respondents at Baseline, Followed Up Between 2000 and 2016

Characteristic	Participants, No. (%)			
	Overall (N = 29 790)	Physical function		ADL disability (n = 4848)
		Healthy (n = 16 494)	Limitations (n = 8448)	
Age, mean (SD), y	63 (11)	61 (10)	65 (11)	68 (13)
Sex				
Female	16 878 (57)	8306 (50)	5494 (65)	3078 (63)
Male	12 912 (43%)	8188 (50)	2954 (35)	1770 (37)
Race and ethnicity				
Hispanic	3371 (11)	1768 (11)	917 (11)	686 (14)
Non-Hispanic Black	5240 (18)	2522 (15)	1592 (19)	1126 (23)
Non-Hispanic White	20 314 (68)	11 689 (71)	5723 (68)	2902 (60)
Education level				
Less than high school	7107 (24)	2917 (18)	2222 (26)	1968 (41)
High school graduate	10 095 (34)	5355 (32)	3209 (38)	1531 (32)
College and above	12 588 (42)	8222 (50)	3017 (36)	1349 (28)
Urbanicity				
Urban	23 976 (80)	13 498 (82)	6661 (79)	3808 (79)
Suburban	5641 (19)	2896 (18)	1734 (21)	1011 (21)
Rural	182 (1)	100 (1)	53 (1)	29 (0.6)
NSES, mean (SD)	0.21 (0.9)	0.12 (0.91)	0.30 (0.81)	0.32 (0.83)
Primary residence ownership	22 496 (76)	13 399 (81)	6252 (74)	2845 (59)
Baseline net wealth, mean (SD), thousands of $	249 (848)	308 (883)	211 (940)	125 (441)
Air pollution concentration, mean (SD)				
PM_2.5_, µg/m^3^	11.3 (2.7)	11.2 (2.8)	11.3 (2.7)	11.5 (2.9)
PM_10-2.5_, µg/m^3^	9.7 (4.9)	9.6 (4.9)	9.7 (4.9)	9.9 (5.0)
NO_2_, ppb	10.7 (6.3)	10.6 (6.2)	10.4 (6.3)	11.2 (6.7)
O_3_, ppb	26.9 (4.0)	27.0 (3.7)	26.9 (3.5)	26.5 (3.6)

Abbreviations: NSES, neighborhood socioeconomic status; ppb, parts per billion.

The 10-year mean (SD) concentrations of air pollution at respondent homes preceding participants’ baseline exams were 11 (3) µg/m^3^ for PM_2.5_, 10 (5) µg/m^3^ for PM_10-2.5_, 11 (6) parts per billion (ppb) for NO_2_, and 27 (4) ppb for O_3_. Air pollution levels were slightly higher among respondents who reported physical function limitations and disability, but these individuals had lower levels of O_3_ compared with those who reported healthy physical function (Table). This aligns with the correlation between the pollutants (eFigure 2 in Supplement 1), where PM_2.5_ and NO_2_ were positively correlated (*r* = 0.6), while O_3_ was negatively correlated with PM_2.5_ (*r* = −0.5) and NO_2_ (*r* = −0.6).

**Figure 2.**
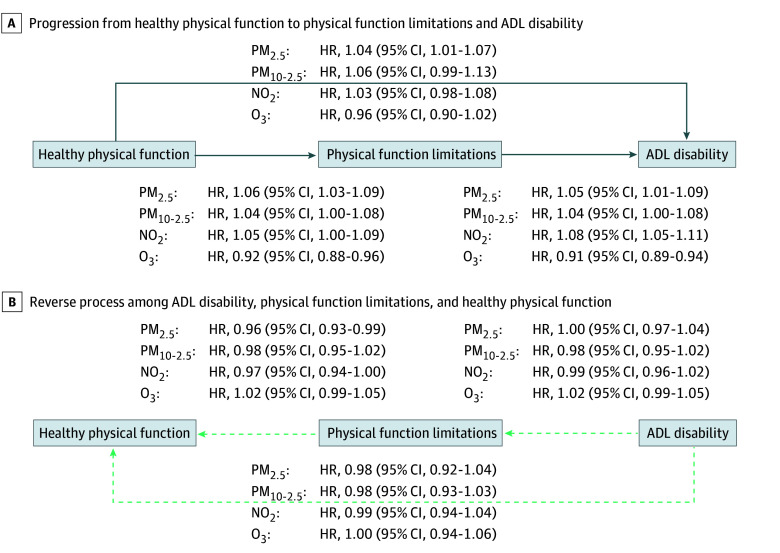
Single-Pollutant Model of Associations of Air Pollution With Transitions to Less and More Severe States of Physical Disability, Health and Retirement Study (2000-2016) Data from 29 790 participants were included in this model. Effect estimates are expressed as hazard ratios (HRs) with 95% CIs per IQR-increment in 10-year average air pollutant concentration. The multistate models adjusted for baseline age, sex, race and ethnicity, years in study, calendar year of examination, education level, baseline net wealth, primary residence ownership, neighborhood socioeconomic status, urbanicity, and a spatial-basis spline (*df* = 10). The primary aim of the study is to identify the impact of air pollution on physical disability process, with death as the absorbing state; therefore, only transition HRs between healthy physical function, physical function limitations, and activities of daily living (ADL) disability are presented. NO_2_ indicates nitrogen dioxide; O_3_, ozone; PM_2.5_, particulate matter with a diameter of 2.5 µm or less; and PM_2.5-10_, particulate matter with a diameter between 2.5 and 10 µm.

**Figure 3.**
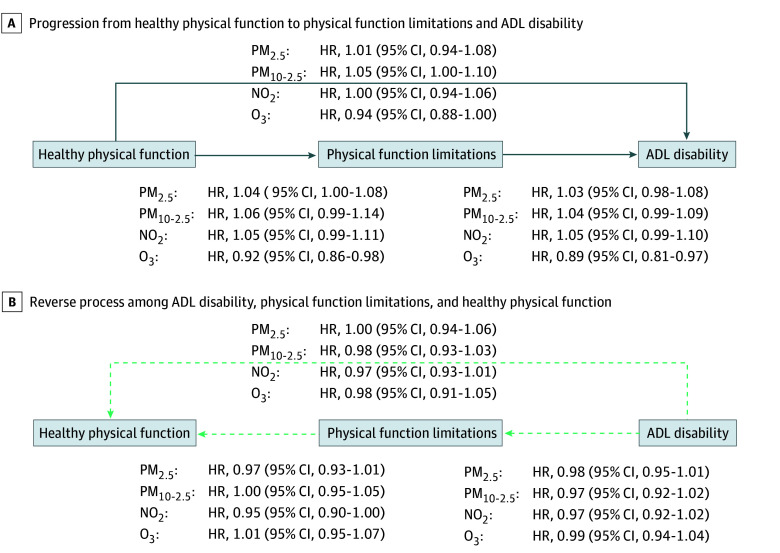
Multipollutant Model of Associations of Air Pollution With Transitions to Less and More Severe States of Physical Disability, Health and Retirement Study (2000-2016) Data from 29 790 participants were included in this model. Effect estimates are expressed as hazard ratios (HRs) with 95% CIs per IQR-increment in 10-year average air pollutant concentration. The multistate models adjusted for baseline age, sex, race and ethnicity, years in study, calendar year of examination, education level, baseline net wealth, primary residence ownership, neighborhood socioeconomic status, urbanicity, a spatial-basis spline (*df* = 10), and all copollutants. The primary aim of the study is to identify the impact of air pollution on physical disability process, with death as the absorbing state; therefore, only transition HRs between healthy physical function, physical function limitations, and activities of daily living (ADL) disability are presented. NO_2_ indicates nitrogen dioxide; O_3_, ozone; PM_2.5_, particulate matter with a diameter of 2.5 µm or less; and PM_2.5-10_, particulate matter with a diameter between 2.5 and 10 µm.

In our multistate modeling, higher PM_2.5_, PM_10-2.5_, and NO_2_ concentrations were associated with greater hazards of transitioning from a state of no physical function limitations to any physical function limitation and ADL disability. We also found evidence that higher concentrations of some of these pollutants were associated with lower probabilities of transitioning toward no physical function limitations and no ADL disability. In contrast, we observed that higher levels of O_3_ were associated with lower hazards of transitioning to more severe physical function limitations (eg, transition from healthy physical function to physical function limitation: hazard ratio [HR], 0.92; 95% CI, 0.86-0.98; to ADL disability: HR, 0.89; 95% CI, 0.81-0.97). These associations were generally stronger in our single-pollutant models (Figure 2) than the multipollutant models (Figure 3) (eg, PM_2.5_ and transition from healthy physical function to physical function limitation, single pollutant model: HR, 1.06; 95% CI, 1.03-1.09; multipollutant model: HR, 1.04; 95% CI, 1.00-1.08), and some estimates were imprecise enough that they could not be statistically distinguished from no association. For example, in the single-pollutant model, NO_2_ was associated with a higher risk of transition from healthy physical function to physical function limitation (HR, 1.05; 95% CI, 1.00-1.09). In the multipollutant model, the HR remained 1.05, but the 95% CI contained the null (95% CI, 0.99-1.11).

**Figure 4.**
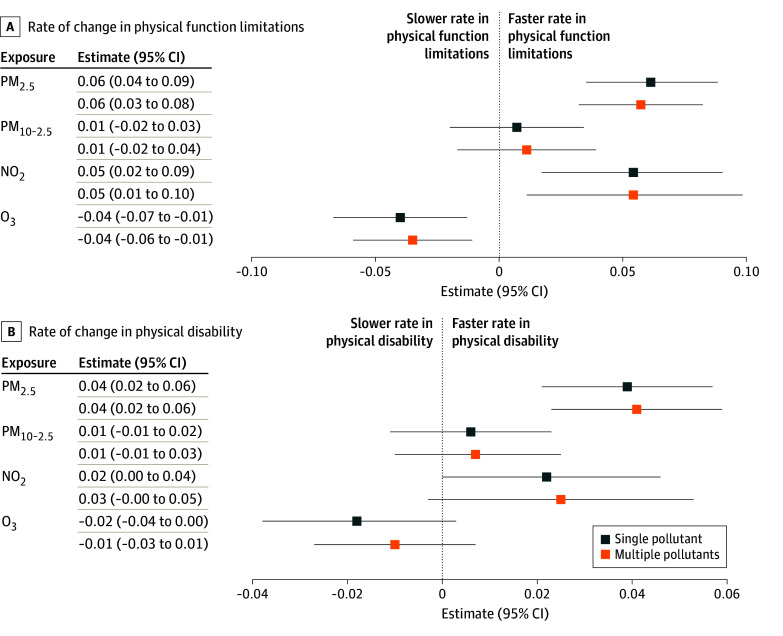
Air Pollution and Rate of Change in Physical Function Limitations and Activities of Daily Living (ADL) Disability in the Health and Retirement Study (2000-2016) Data from 29 790 participants were included in this model. Rate of change is measured in units per year. Effect estimates and 95% CIs are scaled per IQR-increment in 10-year average air pollutant concentration. The general estimating equations were adjusted for baseline age, sex, race and ethnicity, years in study, calendar year of examination, education level, baseline net wealth, primary residence ownership, neighborhood socioeconomic status, urbanicity, a spatial-basis spline (*df* = 10), and a cross-product between years in study and covariates. NO_2_ indicates nitrogen dioxide; O_3_, ozone; PM_2.5_, particulate matter with a diameter of 2.5 µm or less; and PM_2.5-10_, particulate matter with a diameter between 2.5 and 10 µm.

In our secondary analyses testing associations between air pollution and rate of change in physical function limitations and ADL disability during follow-up, the findings were largely comparable with those of the multistate models. A 1-IQR increase in PM_2.5_, PM_10-2.5_, and NO_2_ was associated with a faster development of physical function limitations and ADL disability (Figure 4). The strongest association was observed for PM_2.5_, where a 1-IQR increase was associated with a 0.06–unit/y (95% CI, 0.04-0.09 unit/y) faster progression in physical function limitations. Conversely, higher levels of O_3_ were associated with a slower rate of increase in physical function limitations and ADL disability over time. Again, most observed associations were robust to accounting for potential confounding by copollutants, with the notable exception of O_3_ with ADL disability declines (Figure 4).

In our sensitivity analysis, the multistate model results were robust to restricting our population to healthy respondents at baseline (eFigure 3 in Supplement 1). When refitting our models with different exposure windows, the observed associations showed slight attenuation for the shorter pollution averaging times for both the multistate and GEE models (eFigures 4-6 in Supplement 1).

## Discussion

Over an average follow-up period of 8 years within this nationally representative cohort, higher residential concentrations of outdoor PM_2.5_, PM_10-2.5_, and NO_2_ were associated with higher rates of transitioning from a state of no physical function limitations to states with physical function limitations and ADL disability. Higher concentrations of these pollutants were also, in some instances, associated with lower likelihoods of transitioning from physical function limitations back to a state without physical function limitations. Consistent with our primary findings, we also found that higher residential concentrations of outdoor PM_2.5_ and NO_2_ were associated with faster progression of continuous measures of physical function limitations and ADL disability. Notably, higher levels of O_3_ appeared to be associated with fewer physical function limitations and less ADL disability that could not be explained entirely by negative correlations with the other pollutants. Overall, these findings have potential clinical and societal significance as the management of physical disability costs the government an estimated $400 billion annually.^29^

Our study contributes to the literature by comprehensively investigating the impacts of several key air pollutants on the pattern of progression in physical function limitations and ADL disability in later life in the same analysis. While some studies have assessed the role of air pollution on ADL disability, less attention has been paid to physical function limitations,^30,31^ which represent the intermediate state of physical disability. This analysis builds on our own past work in the HRS,^17^ which also showed higher risks of ADL disability with higher PM_2.5_, PM_10-2.5_, and NO_2_ concentrations and lower concentrations of O_3_. We extend those findings by now documenting that these pollutants were independently associated with greater risks of transitioning from no physical function limitations to states with physical function limitations and subsequentially ADL disability as well as lower likelihoods of reverting back to states without physical function limitations. These findings are in line with our current understanding of the physical disability process, suggesting that air pollution likely exerts its influence steadily on the body over a longer duration. This also raises the notable point that higher levels of air pollution contribute to individuals with physical function limitations progressing to more severe ADL disability, suggesting that there are opportunities for interventions even after the initial manifestation of the functional limitations in late life.

Although this work is novel in its simultaneous evaluation of both physical function limitations and ADL disability, our findings are generally consistent with literature on these types of outcomes. For example, results from the US-based Chicago Healthy and Aging Project also reported that higher NO_2_ levels were associated with faster physical disability progression.^13^ Our observations are also in alignment with research in low- and middle-incomed countries. For example, studies within the Global Aging and Adult Health Study from China, Ghana, India, Mexico, the Russian Federation, and South Africa found that higher levels of PM_2.5_ were associated with significant decreases in grip strength and mobility performance, 2 measures of physical function limitations based on upper and lower body muscle strength.^31^ Similarly, the China Longitudinal Healthy Longevity Survey (CLHLS) and China Longitudinal Health and Retirement Study (CLHRS) reported associations between higher levels of PM_2.5_ and increased risks of incident ADL disability.^15,16,32^ Our findings are also consistent with previous data on associations of long-term exposure to PM_2.5_, PM_10-2.5_, and NO_2_ with frailty,^33,34,35^ a summary index reflecting the vulnerability of older adults in terms of physical function limitations, ADL disability, and multimorbidity conditions. For example, a study derived from CLHLS found a 10 µg/m^3^ increase in PM_2.5_ was associated with a 5% greater risk of frailty.^33^ Cross-sectional studies of older adults in Korea and the United Kingdom also reported that higher PM_2.5_, PM_10-2.5_, NO_2_, and O_3_ levels were associated with a higher risk of having prefrailty or frailty.^34,35^ Together, these studies from aging populations around the world provide evidence to support the hypothesis that higher air pollution levels are associated with greater risks of physical disability in late life.

Interestingly, we found that higher PM_2.5_ and NO_2_ concentrations had HRs less than 1 for transitioning from a state with physical function limitations to a state without these limitations. Although these findings were not always as large as some of the corresponding ones for this pollutant with transition to more severe states, it is possible that inflammation caused by air pollution might slow the body’s ability to recover from transient episodes of physical functional limitations. Additionally, physical function limitations are a common consequence of decreased respiratory function. Since air pollution is a known risk factor for respiratory disease, it is plausible that higher levels of exposure also make it more difficult to recover from physical function limitations due to decreased or impaired lung function. This is most plausible for people with mild physical function restrictions.^36,37^

Contrary to our hypothesis, we found that higher levels of O_3_ were associated with a higher likelihood of transitions to a state of no physical function limitations and to greater physical function improvements over time. Notably, this is not the first study to find seemingly protective associations with O_3_. In fact, conclusions regarding the impacts of O_3_ on health are quite mixed. While some studies based on data from China, US, and Korea demonstrated positive associations between O_3_ and adverse health outcomes, including declines in cognitive function, lung function, and frailty,^34,38,39^ numerous other studies have reported either null or seemingly protective associations between O_3_ and health.^40,41,42^ For example, a cohort study from the UK found associations between higher levels of O_3_ and lower rates of all-cause mortality,^40^ and our research in the HRS showed evidence of higher levels of O_3_ associated with lower risks of dementia and physical disability.^17,43^ Although it is possible that there is some unmeasured confounding present in this study, the observed association with O_3_ could not be explained entirely by either its negative correlation with other pollutions or by a lack of adjustment of spatial variables. Instead, we speculate that low levels of O_3_ may reflect locations where O_3_ reacts with other pollutants to form new, but unmeasured, toxic components that increase the risk of physical function limitations and ADL disability. Notably, emerging studies indirectly support this hypothesis with evidence from roadside studies in Germany and Canada suggesting that there are traffic-related amine emissions that rapidly react with acids to form secondary organic aerosols in near-road, low O_3_ conditions.^44,45^ However, further research is needed to directly validate this hypothesis.

### Strengths and Limitations

Our study has several strengths. First, it is a large longitudinal study that assessed the association between several key air pollutants in relation to the dynamic progression of physical disability. Our use of a multistate model allowed us to consider several states of physical disability simultaneously in one model to better understand whether air pollution was independently associated with transitions between each physical disability state and consider both declines and recovery. We also explicitly accounted for associations with death as a competing risk. Second, unlike previous studies that only estimated 1-year or 3-year average air pollution levels, we used state-of-the-art models to estimate 10-year average air pollution levels. This is important given that physical function limitation and disability progression is a longer-term process, unfolding over many years. Notably, our results showed that associations for 10-year average exposures were stronger than those of 1-year and 5-year average exposures, providing support for this point. Finally, our study used data from the HRS, a nationally representative cohort with a large sample size, high response and follow-up rate, and rigorous information on personal- and neighborhood-level confounders. We also include proxy respondents from the interview. These help to ensure that our results were robust to bias and have strong generalizability to the US population.

Despite these many strengths, our study also has limitations. First, we only studied residential outdoor air pollution concentrations and did not consider indoor concentrations or time spent away from home. This could introduce measurement error into our analysis. However, since there is typically a high positive correlation between outdoor and indoor air pollution, with more than 75% of indoor pollution variation explained by outdoor variation,^46,47^ and many participants are retired, it is unlikely that such measurement error significantly affected our observed associations. Outdoor air pollution is also what is regulated by environmental policy. Second, although the multistate model accounted for attrition due to death, there still is the possibility that those most susceptible to air pollution did not survive to the start of our study. Relatedly, it is possible that people who develop physical function limitations and disability are more likely to be lost to follow-up. To address this possibility for selection bias, we adjusted for participant selection into the study and loss after follow-up by using survey weights, so we do not expect this to be a large source of bias. Nonetheless, if there was a healthy retention effect in our cohort, this would most likely bias our results toward the null. We also did not include information from the exit interview, which took place within 1 year after a respondent’s death because it only included ADL questions. As a result, we cannot rule out the possibility that we may have missed individuals who reported severe physical function limitations and ADL disability before death. However, we believe that this bias would likely be toward the null and not substantially alter our conclusion as we used death as the absorbing state in the model. Additionally, because the HRS mainly contains self-reported indicators rather than clinical diagnosis of diseases, we did not explore effect modification by diseases that influence physical function, such as respiratory disease and cardiovascular disease; we note that this would be an interesting line of research in the future.

## Conclusions

In this nationwide cohort study, we found evidence that long-term exposure to ambient PM_2.5_, PM_10-2.5_, and NO_2_, but not O_3_, were associated with greater risks of progressing from no physical function limitations to states of more physical function limitations and ADL disability; long-term exposure to PM_2.5_ was associated with a lower risk of reverting back to better physical function. Our results suggest that reducing certain key air pollutants may delay the onset of physical function limitations and ADL disability, as well as lessen the severity of physical disability even after the process begun, thereby shedding light on future regulations and policies in targeting interventions to control health care expenses and achieve the goal of healthy aging.

## Notes

Supplement 1. **eFigure 1.** Flowchart of the Analytic Study Population Selection **eFigure 2.** Pearson Correlation Between 4 Key Air Pollutants **eFigure 3.** Single-Pollutant Associations of Air Pollution With Transition to More Severe and Less Severe States of Physical Disability Among Those With Healthy Physical Function at Baseline, Health and Retirement Study **eFigure 4.** Single Pollutant Associations of Air Pollution With Transition to More Severe and Less Severe States of Physical Disability Using a Shorter Averaging Period, Health and Retirement Study **eFigure 5.** Single-Pollutant Associations of Air Pollution With Transition to More Severe and Less Severe States of Physical Disability Using the Shortest Averaging Period, Health and Retirement Study **eFigure 6.** Associations Between an IQR-Increment in Air Pollution of Different Exposure Windows and Rate of Change in Physical Function Limitation and ADL Disability in the Health and Retirement Study **eTable.** Demographic Characteristics for Health and Retirement Study Respondents Excluded From the Study and Included in the Study

Supplement 2.
Data Sharing Statement
