# Supplementary material for: Air Pollution and the Progression of Physical Function Limitations and Disability in Aging Adults
Source: JAMA Netw Open. 2026 Feb 11;9(2):e2558699. doi: 10.1001/jamanetworkopen.2025.58699 (PMC12895286; doi:10.1001/jamanetworkopen.2025.58699)
Supplement: Supplement 1. — eFigure 1. Flowchart of the Analytic Study Population Selection eFigure 2. Pearson Correlation Between 4 Key Air Pollutants eFigure 3. Single-Pollutant Associations of Air Pollution With Transition to More Severe and Less Severe States of Physical Disability Among Those With Healthy Physical Function at Baseline, Health and Retirement Study eFigure 4. Single Pollutant Associations of Air Pollution With Transition to More Severe and Less Severe States of Physical Disability Using a Shorter Averaging Period, Health and Retirement Study eFigure 5. Single-Pollutant Associations of Air Pollution With Transition to More Severe and Less Severe States of Physical Disability Using the Shortest Averaging Period, Health and Retirement Study eFigure 6. Associations Between an IQR-Increment in Air Pollution of Different Exposure Windows and Rate of Change in Physical Function Limitation and ADL Disability in the Health and Retirement Study eTable. Demographic Characteristics for Health and Retirement Study Respondents Excluded From the Study and Included in the Study [file jamanetwopen-e2558699-s001.pdf]

Supplemental Online Content

Gao J, de Leon CM, Szpiro A, et al. Long-term air pollution and physical function limitations and disability in aging adults. *JAMA Netw Open.* 2026;9(2):e2558699. doi:10.1001/jamanetworkopen.2025.58699

eFigure 1. Flowchart of the Analytic Study Population Selection

eFigure 2. Pearson Correlation Between 4 Key Air Pollutants

eFigure 3. Single-Pollutant Associations of Air Pollution With Transition to More Severe and Less Severe States of Physical Disability Among Those With Healthy Physical Function at Baseline, Health and Retirement Study

eFigure 4. Single-Pollutant Associations of Air Pollution With Transition to More Severe and Less Severe States of Physical Disability Using a Shorter Averaging Period, Health and Retirement Study

eFigure 5. Single-Pollutant Associations of Air Pollution With Transition to More Severe and Less Severe States of Physical Disability Using the Shortest Averaging Period, Health and Retirement Study

eFigure 6. Associations Between an IQR-Increment in Air Pollution of Different Exposure Windows and Rate of Change in Physical Function Limitation and ADL Disability in the Health and Retirement Study

eTable. Demographic Characteristics for Health and Retirement Study Respondents Excluded From the Study and Included in the Study

This supplemental material has been provided by the authors to give readers additional information about their work.

© 2026 Gao J et al. *JAMA Network Open*.

eFigure 1. Flowchart of the Analytic Study Population Selection

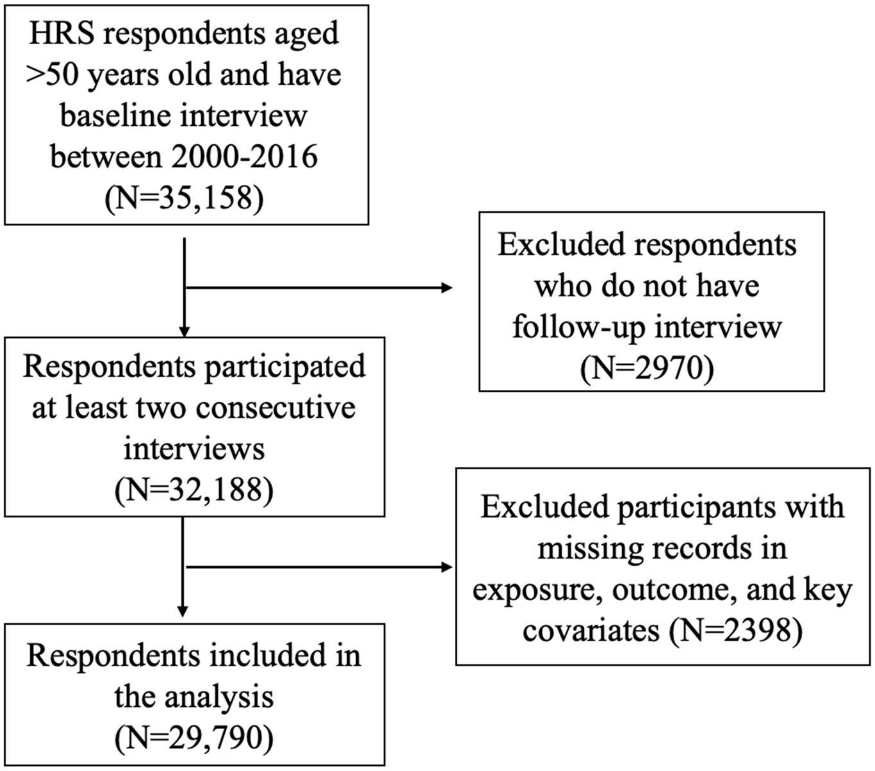

© 2026 Gao J et al. *JAMA Network Open*.

eFigure 2. Pearson Correlation Between 4 Key Air Pollutants

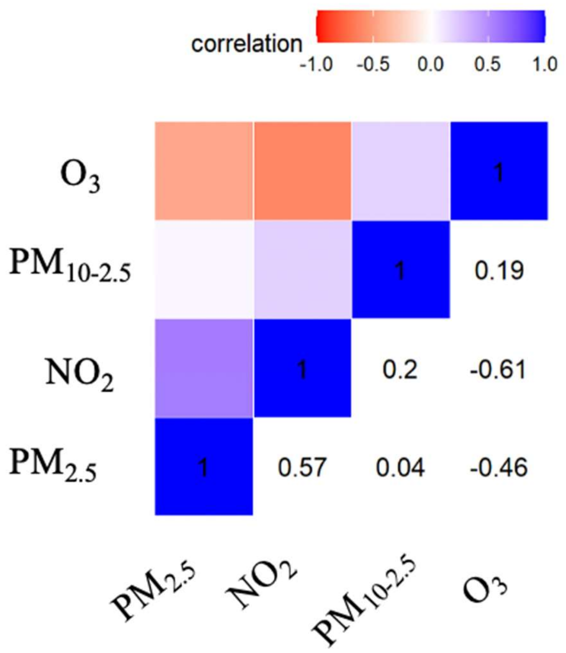

© 2026 Gao J et al. *JAMA Network Open*.

eFigure 3. Single-Pollutant Associations of Air Pollution With Transition to More Severe (A) and Less Severe (B) States of Physical Disability Among Those With Healthy Physical Function at Baseline, Health and Retirement Study (2000-2016, N=16,494)

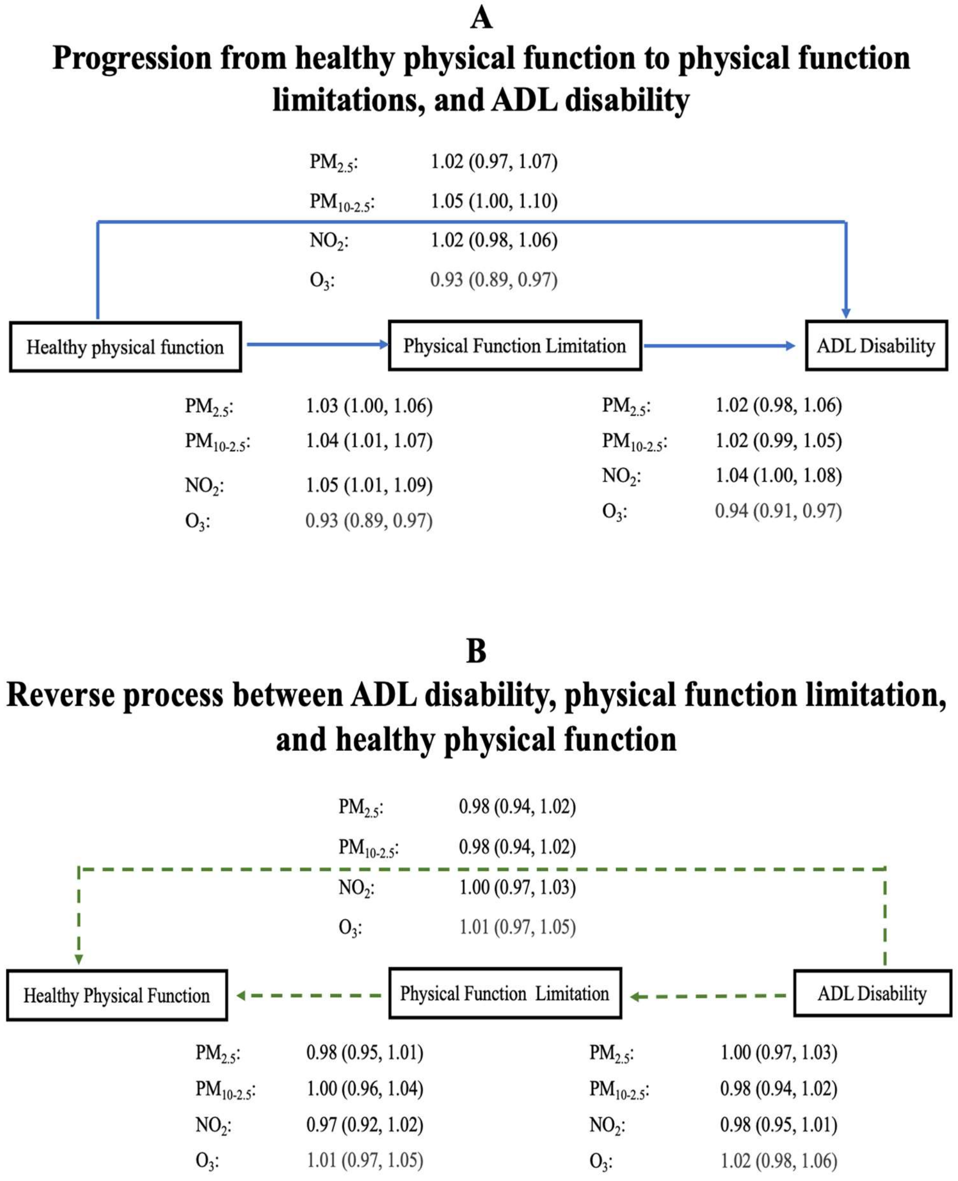

*Effect estimates are expressed as hazard ratios (95% confidential interval) per IQR-increment in 10-year average air pollutant concentration.

*Multistate model adjusted for: baseline age, gender, race/ethnicity, years in study, and calendar year of examination, education level, baseline net wealth, primary residence ownership, neighborhood socio-economic status, urbanicity, and a spatial-basis spline df=10.

© 2026 Gao J et al. *JAMA Network Open*.

eFigure 4. Single-Pollutant Associations of Air Pollution With Transition to More Severe and Less Severe States of Physical Disability Using a Shorter Averaging Period, Health and Retirement Study (2000-2016, N=29,790)

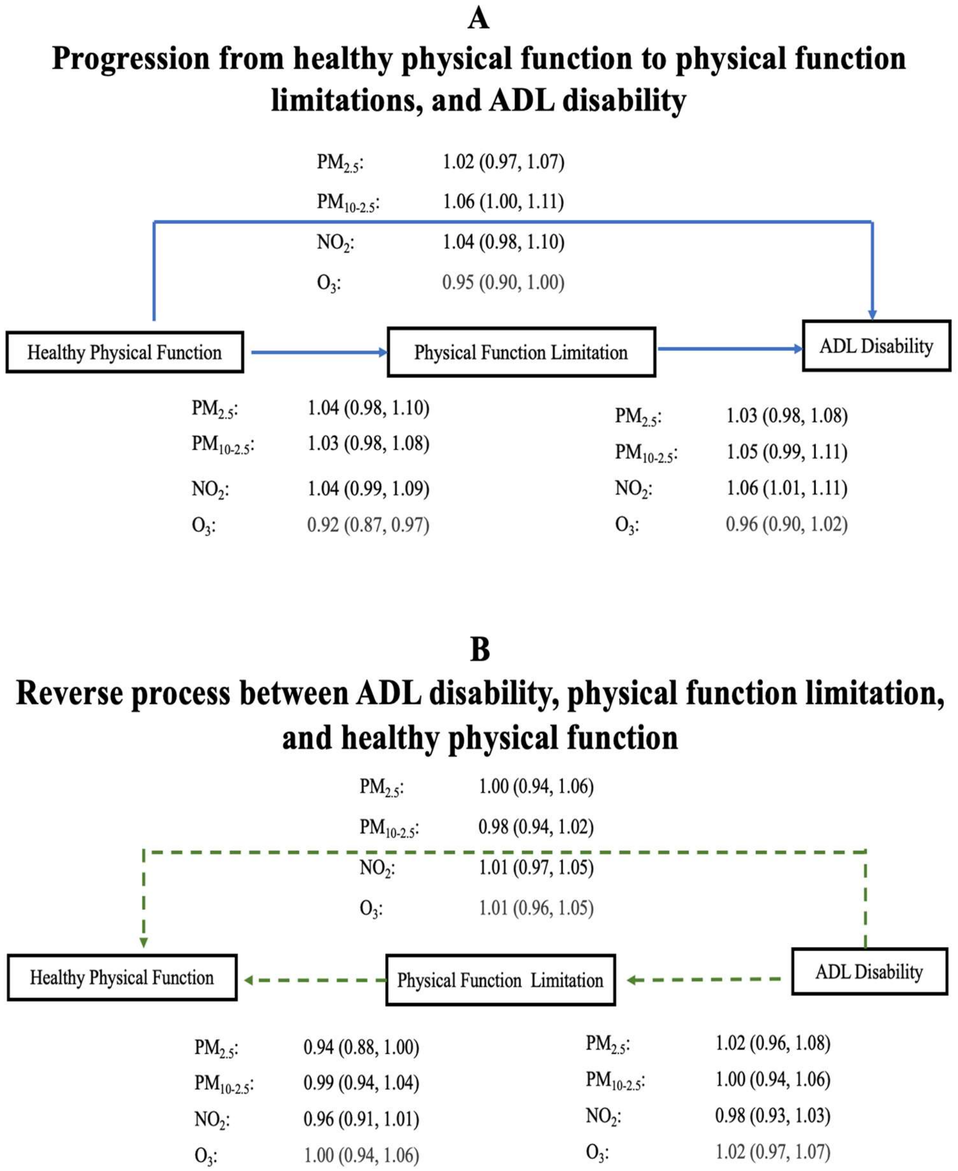

*Effect estimates are expressed as hazard ratios (95% confidential interval) per IQR-increment in 5-year average air pollutant concentration. This differs from the main analysis that uses a 10-year average concentration.

*Multistate model adjusted for: baseline age, gender, race/ethnicity, years in study, and calendar year of examination, education level, baseline net wealth, primary residence ownership, neighborhood socio-economic status, urbanicity, and a spatial-basis spline df=10

© 2026 Gao J et al. *JAMA Network Open*.

eFigure 5. Single-Pollutant Associations of Air Pollution With Transition to More Severe (A) and Less Severe (B) States of Physical Disability Using the Shortest Averaging Period, Health and Retirement Study (2000-2016, N=29,790)

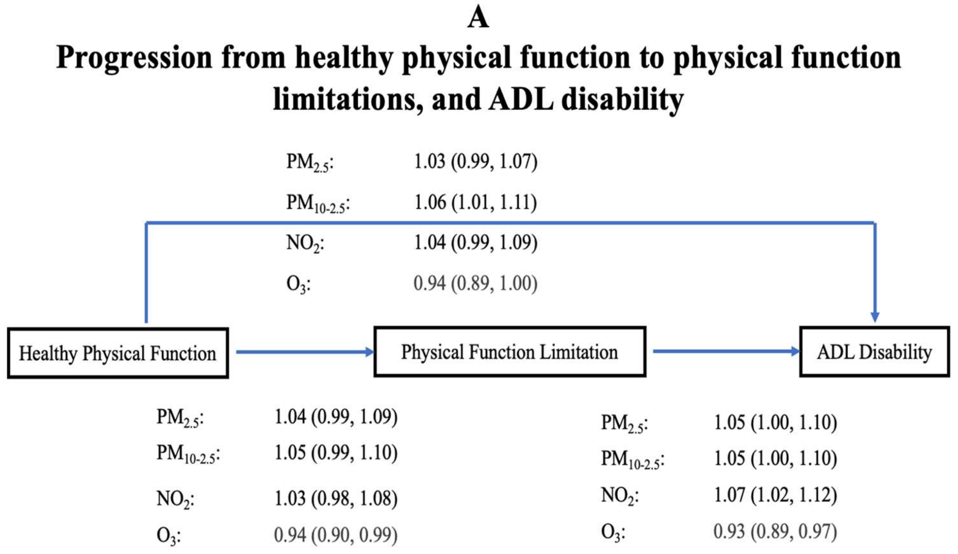

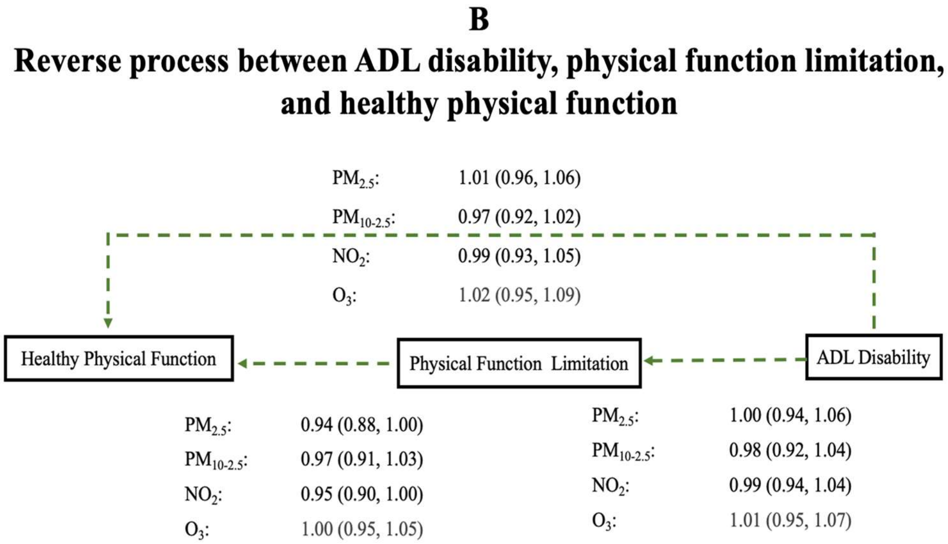

*Effect estimates are expressed as hazard ratios (95% confidential interval) per IQR-increment in 1-year average air pollutant concentration. This differs from the main analysis that uses a 10-year average concentration.

*Multistate model adjusted for: baseline age, gender, race/ethnicity, years in study, and calendar year of examination, education level, baseline net wealth, primary residence ownership, neighborhood socio-economic status, urbanicity, and a spatial-basis spline df=10

© 2026 Gao J et al. *JAMA Network Open*.

eFigure 6. Associations Between an IQR-Increment in Air Pollution of Different Exposure Windows and Rate of Change (Units per Year) in Physical Function Limitation and ADL Disability in the Health and Retirement Study (2000-2016, N=29,790)

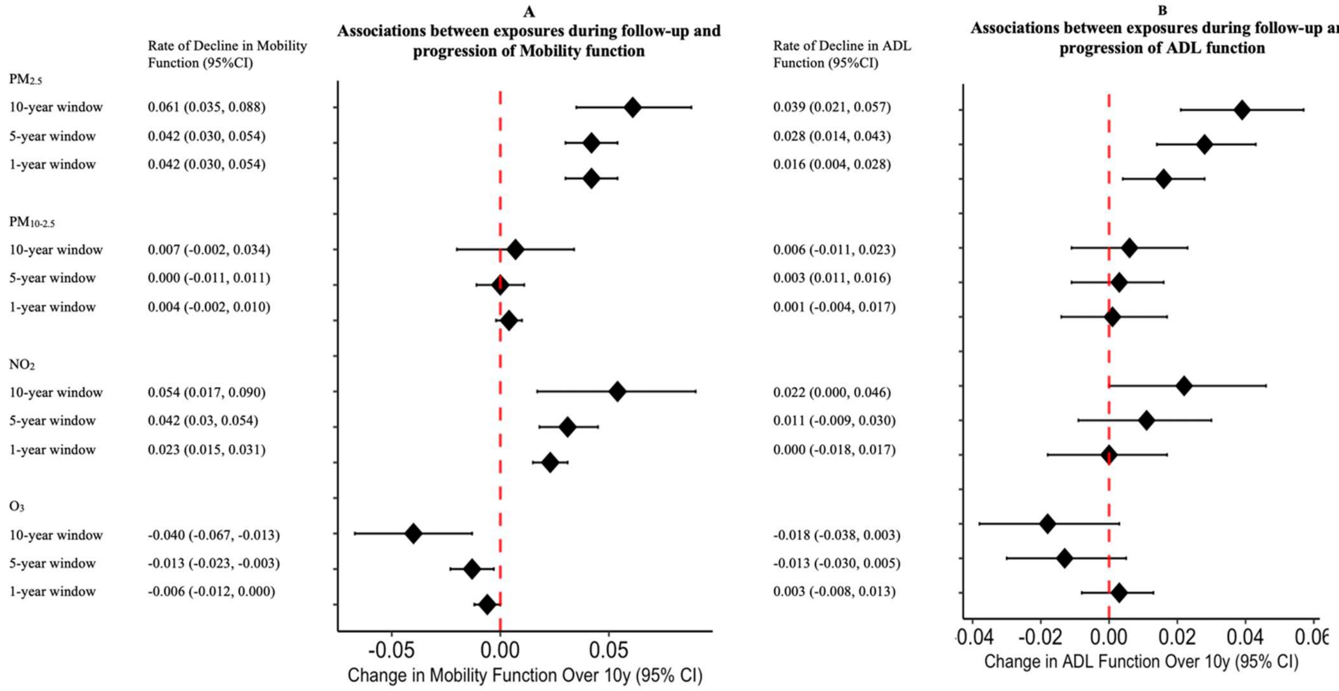

*GEE model, covariates adjusted for: baseline age, gender, race/ethnicity, years in study, and calendar year of examination, education level, baseline net wealth, primary residence ownership, neighborhood socio-economic status, urbanicity, and a spatial-basis spline df=10, and cross product between years in study and covariate

© 2026 Gao J et al. *JAMA Network Open*.

eTable. Demographic Characteristics for Health and Retirement Study Respondents Excluded From the Study and Included in the Study

	Exclude due to missingness (N=2,398) No (%) or mean (SD)	Respondents included in the analysis (N=29,790) No (%) or mean (SD)
Male		
Female	1,294 (54%)	16,878 (57%)
Non-Hispanic White	1,416 (59%)	20,314 (68%)
Non-Hispanic Black	579 (24%)	5,240 (18%)
Hispanic	396 (16%)	3,371 (11%)
High-School and Graduate	871 (36%)	10,095 (34%)
College and Above	933 (39%)	12,588 (42%)
$PM_{2.5}$ (µg/m^3)	11.5 (3.2)	11.3 (2.7)
$PM_{10-2.5}$ (µg/m^3)	9.0 (4.2)	9.7 (4.9)
NO_2 (ppb)	11.4 (7.1)	10.7 (6.3)
O_3 (ppb)	26.4 (4.2)	26.9 (4.0)

© 2026 Gao J et al. *JAMA Network Open*.
