# Supplementary material for: Air Pollution and the Progression of Physical Function Limitations and Disability in Aging Adults
Source: JAMA Netw Open. 2026 Feb 11;9(2):e2558699. doi: 10.1001/jamanetworkopen.2025.58699 (PMC12895286; doi:10.1001/jamanetworkopen.2025.58699)
Supplement: Supplement 2. — Data Sharing Statement [file jamanetwopen-e2558699-s002.pdf]

Data Sharing Statement

Gao. Long-Term Air Pollution and Physical Function Limitations and Disability in Aging Adults. *JAMA Netw Open*. Published February 11, 2026. doi:10.1001/jamanetworkopen.2025.58699

Data
Data available: Yes
Data types: Participant data with identifiers
How to access data: All data is available for request from the Health and Retirement Study as restricted access products. https://hrs.isr.umich.edu/data-products/restricted-data/available-products/9691)
When available: beginning date: 08-01-2025

Supporting Documents
Document types: None

Additional Information
Who can access the data: Data are available for investigators with approved IRB applications and restricted data access applications from the Health and Retirement Study.
Types of analyses: Data are available for analysis per the rules of the Health and Retirement Study and the National Institutes of Aging.
Mechanisms of data availability: After a signed data access agreement with the Health and Retirement Study data are available through a secure enclave.
